# Supplementary material for: Chemical Investigation of Endophytic Diaporthe unshiuensis YSP3 Reveals New Antibacterial and Cytotoxic Agents
Source: J Fungi (Basel). 2023 Jan 19;9(2):136. doi: 10.3390/jof9020136 (PMC9963169; doi:10.3390/jof9020136)
Supplement: Supplementary file 1 [file jof-09-00136-s001.zip › jof-2103647-supplementary.pdf]

Supporting Information

Chemical investigation of endophytic *Diaporthe unshiuensis* YSP3 reveals new antibacterial and cytotoxic agents

Babar Khan [1,2,†], Yu Li [1,2,†], Wei Wei [3], Guiyou Liu [3], Cheng Xiao [1,2], Bo He [1,2], Chen Zhang [1,2], Nasir Ahmed Rajput [4], Yonghao Ye [1,2,*], Wei Yan [1,2,*]

[1] College of Plant Protection, State & Local Joint Engineering Research Center of Green Pesticide Invention and Application, Nanjing Agricultural University, Nanjing 210095, P. R. China

[2] Key Laboratory of Integrated Management of Crop Diseases and Pests, Ministry of Education, Nanjing 210095, P. R. China

[3] School of Life Sciences and Chemical Engineering, Jiangsu Second Normal University, Nanjing, 211200, P. R. China

[4] Department of Plant Pathology, University of Agriculture, Faisalabad, 38000, Pakistan

[*] Correspondence: yeyh@njau.edu.cn (Y.Y.), yanwei@njau.edu.cn (W.Y.); Tel: +86-25-84399753

[†] These authors contributed equally to this paper

Contents

Table S1. X-ray crystallographic data for **1**
Table S2. X-ray crystallographic data for **2**
Table S3. The inhibition rates of **1** against normal cells LO2 and HaCaT
Figure S1. Phylogenetic analysis of *Diaporthe unshiuensis* YSP3
Figure S2. The ^{1}H-NMR spectrum of **1** in acetone-d_6
Figure S3. The ^{13}C-NMR spectrum of **1** in acetone-d_6
Figure S4. The HSQC spectrum of **1** in acetone-d_6
Figure S5. The HMBC spectrum of **1** in acetone-d_6
Figure S6. The^{1}H-^{1}H COSY spectrum of **1** in acetone-d_6
Figure S7. The NOESY spectrum of **1** in acetone-d_6
Figure S8. The DEPT spectrum of **1** in acetone-d_6
Figure S9. The ^{1}H-NMR spectrum of **2** in $CDCl_3$
Figure S10. The ^{13}C-NMR spectrum of **2** in $CDCl_3$
Figure S11. The HSQC spectrum of **2** in $CDCl_3$
Figure S12. The HMBC spectrum of **2** in $CDCl_3$
Figure S13. The^{1}H-^{1}H COSY spectrum of **2** in $CDCl_3$
Figure S14. The NOESY spectrum of **2** in $CDCl_3$
Figure S15. The ^{1}H-NMR spectrum of **3** in acetone-d_6
Figure S16. The ^{13}C-NMR spectrum of **3** in acetone-d_6
Figure S17. The HSQC spectrum of **3** in acetone-d_6
Figure S18. The HMBC spectrum of **3** in acetone-d_6
Figure S19. The^{1}H-^{1}H COSY spectrum of **3** in acetone-d_6
Figure S20. The NOESY spectrum of **3** in acetone-d_6
Figure S21. The DEPT spectrum of **3** in acetone-d_6
Figure S22. The ^{1}H-NMR spectrum of **4** in acetone-d_6
Figure S23. The ^{13}C-NMR spectrum of **4** in acetone-d_6
Figure S24. The HSQC spectrum of **4** in acetone-d_6
Figure S25. The HMBC spectrum of **4** in acetone-d_6
Figure S26. The^{1}H-^{1}H COSY spectrum of **4** in acetone-d_6
Figure S27. The NOESY spectrum of **4** in acetone-d_6
Figure S28.The DEPT spectrum of **4** in acetone-d_6
Figure S29. ^{1}H NMR of *S*-MTPA spectrum of **4** in acetone-d_6
Figure S30. ^{1}H NMR of *R*-MTPA spectrum of **4** in acetone-d_6

Table S1. X-ray crystallographic data for **1.**

Empirical formula	$C_{15}H_{16}O_7$
Formula weight	308.29
Temperature/K	296(2)
Crystal system	triclinic
Space group	P-1
a/Å	8.2397(3)
b/Å	8.5629(3)
c/Å	11.4399(4)
α/°	69.676(2)
β/°	86.302(2)
γ/°	75.660(2)
Volume/Å^3	733.11(5)
Z	2
ρ_{calc}g/cm^3	1.478
μ/mm^{-1}	1.035
F(000)	344.0
Crystal size/mm^3	0.12 × 0.11 × 0.09
Radiation	CuKα (λ = 1.54178)
2Θ range for data collection/ °	11.086 to 129.992
Index ranges	-8 ≤ h ≤ 9, -10 ≤ k ≤ 8, -13 ≤ l ≤ 13
Reflections collected	4349
Independent reflections	2407 [R_{int} = 0.0183, R_{sigma} = 0.0264]
Data/restraints/parameters	2407/0/213
Goodness-of-fit on F^2	1.071
Final R indexes [I>=2σ (I)]	R_1 = 0.0355, wR_2 = 0.1007
Final R indexes [all data]	R_1 = 0.0374, wR_2 = 0.1024

Table S2. X-ray crystallographic data for **2.**

Empirical formula	$C_{15}H_{18}O_7$
Formula weight	310.306
Temperature/K	296.15
Crystal system	monoclinic
Space group	$P2_1/c$
a/Å	11.3791(11)
b/Å	7.4153(6)
c/Å	16.9366(15)
α/°	90
β/°	108.048(5)
γ/°	90
Volume/Å^3	1358.8(2)
Z	4
ρ_{calc}g/cm^3	1.517
μ/mm^{-1}	1.027
F(000)	658.6
Crystal size/mm^3	0.12 × 0.11 × 0.09
Radiation	Cu Kα (λ = 1.54178)
2Θ range for data collection/ °	8.18 to 136.36
Index ranges	-13 ≤ h ≤ 9, -8 ≤ k ≤ 8, -19 ≤ l ≤ 20
Reflections collected	5641
Independent reflections	2426 [R_{int} = 0.0213, R_{sigma} = 0.0254]
Data/restraints/parameters	2426/0/205
Goodness-of-fit on F^2	1.074
Final R indexes [I>=2σ (I)]	R_1 = 0.0374, wR_2 = 0.1092
Final R indexes [all data]	R_1 = 0.0393, wR_2 = 0.1118
Largest diff. peak/hole / e Å^{-3}	0.25/-0.18

Table S3. The inhibition rates of **1** against normal cells LO2 and HaCaT

Compounds		Inhibition rates	
Concentration		LO2	HaCaT
Phomopthane A (**1**)	100 μM	9.453±1.499	8.483±1.366
	50 μM	4.361±0.775	4.652±2.052
	25 μM	1.923±3.714	1.733±3.424
	12.5 μM	2.627±1.818	1.551±2.018
	6.25 μM	-0.894±2.336	-1.247±3.793
	3.125 μM	-2.329±3.015	-1.976±0.961
	1.56 μM	0.433±2.160	-2.645±3.334
	0.78 μM	-3.440±1.015	-4.105±2.477
Taxol [a]	10 μM	91.278±1.766	92.004±0.139

[a] Positive control

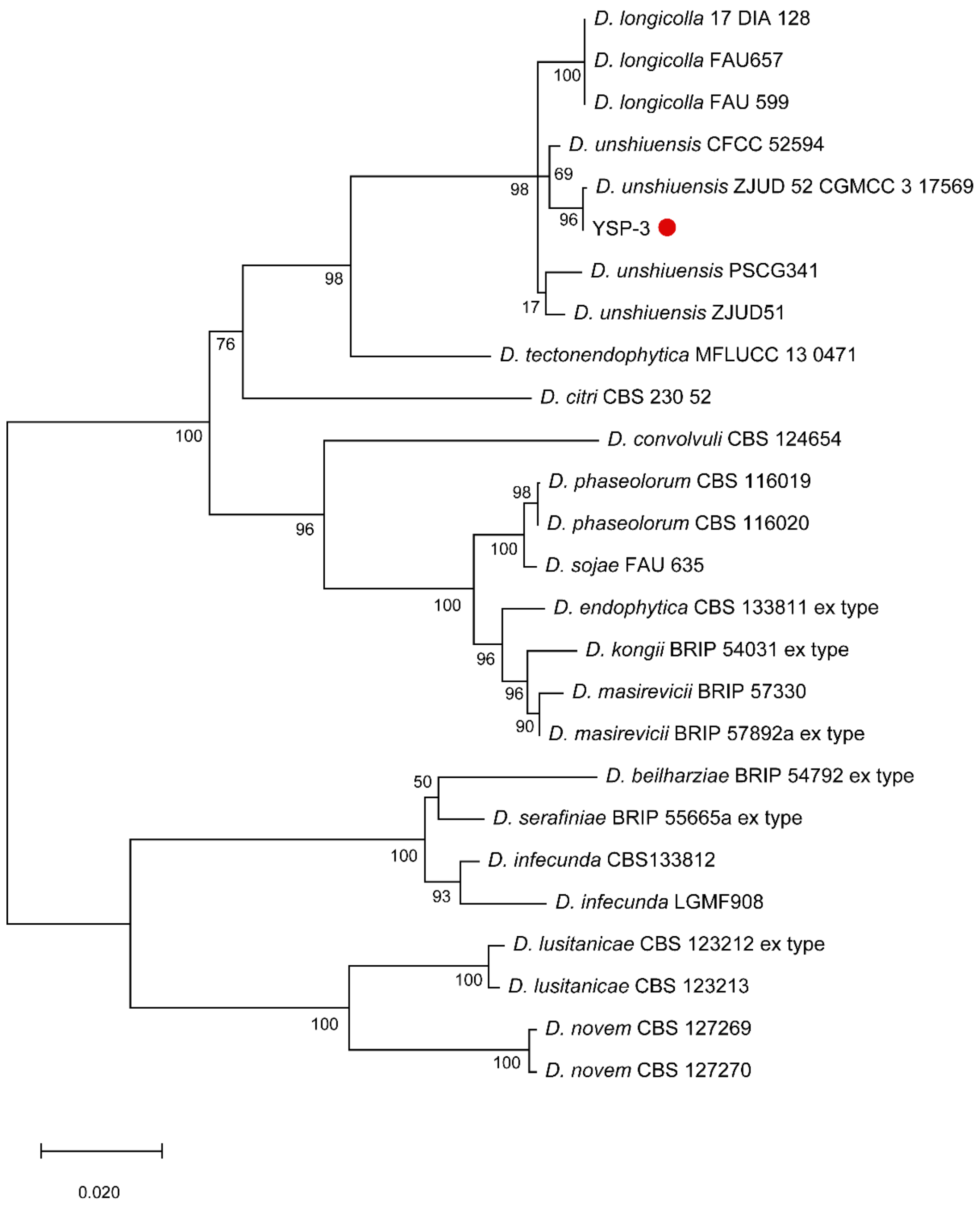

Figure S1. Phylogenetic analysis of *Diaporthe unshiuensis* YSP3.

For phylogenetic analysis, additional reference sequences of 25 strains from 15 representative *Diaporthe* spp. that had been identified were retrieved from GenBank. Multiple sequence alignments were generated using the MUSCLE program implemented in the MEGA software (version 5.2.2), and the concatenated alignment was generated by fusing the ITS (GenBank OP804247), TEF1 (OP884094), HIS (OP884093), CAL (OP884092), and TUB (OP884095) sequences using Phylosuite-1.2.1. Phylogenetic trees were constructed for the concatenated sequences using a maximum-likelihood method, a robust test of 1,000 bootstraps.

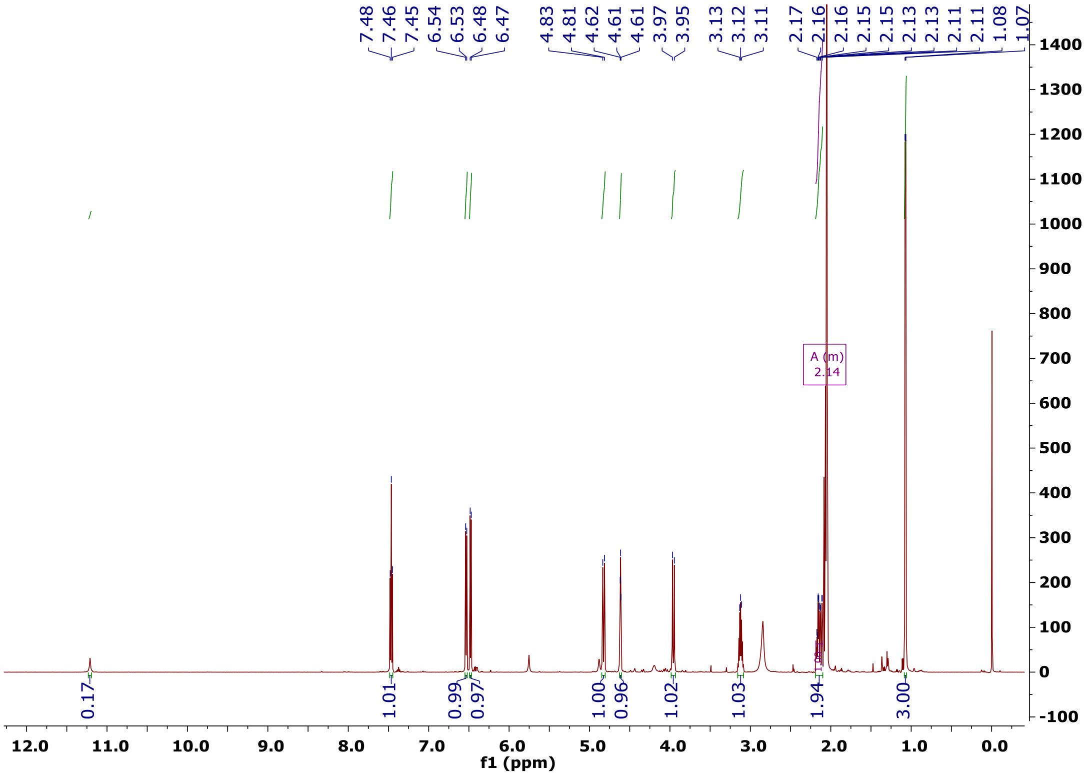

Figure S2. ^{1}H NMR spectrum of **1** (acetone-d_6, 600 MHz).

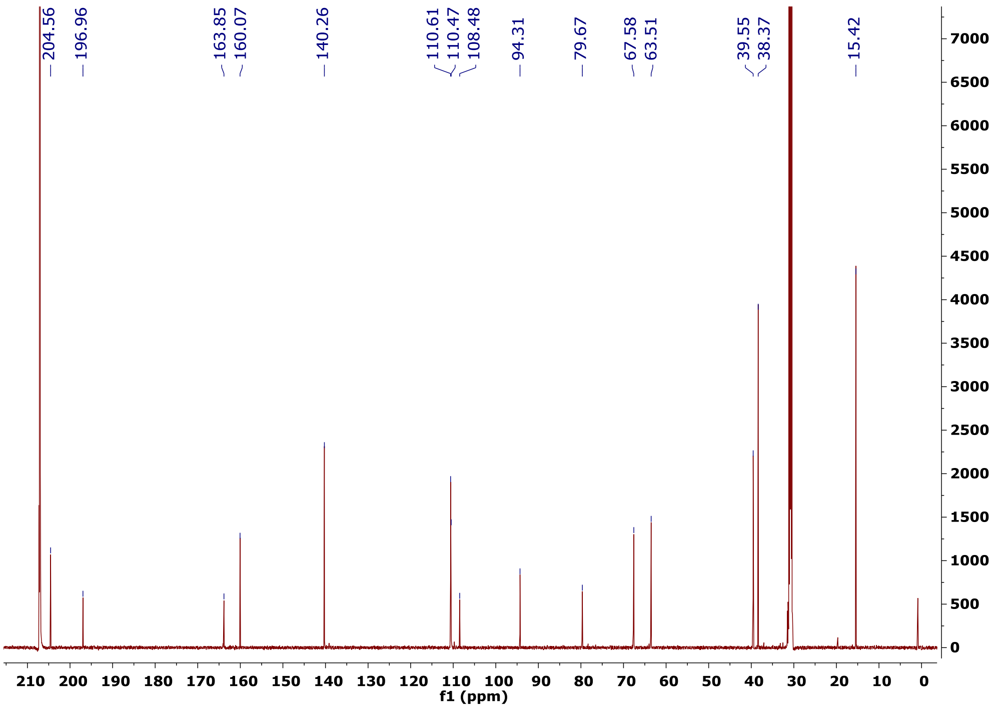

Figure S3. ^{13}C NMR spectrum of **1** (acetone-d_6, 150 MHz).

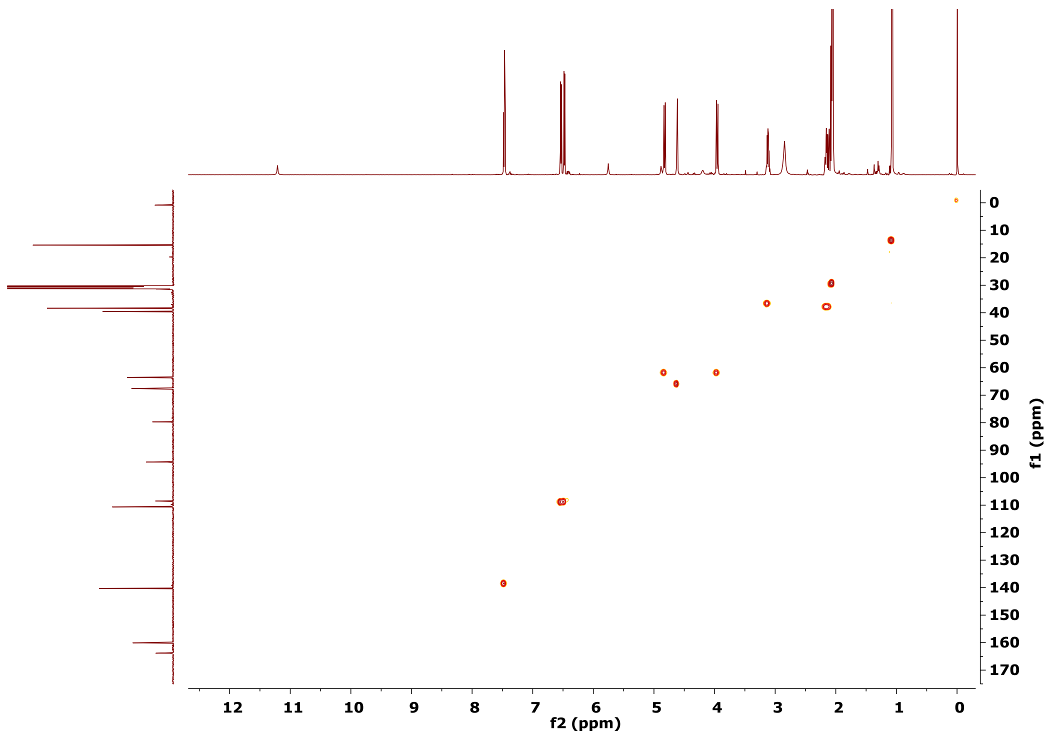

Figure S4. HSQC spectrum of **1** (acetone-d_6, 600 MHz).

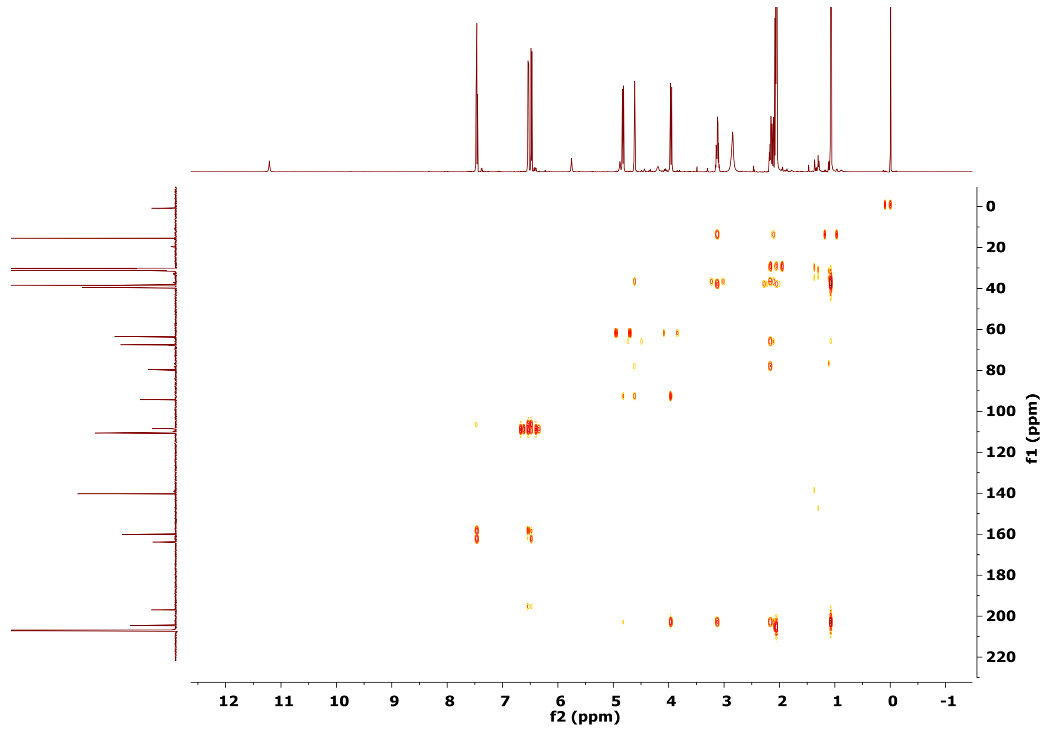

Figure S5. HMBC spectrum of **1** in acetone-d_6.

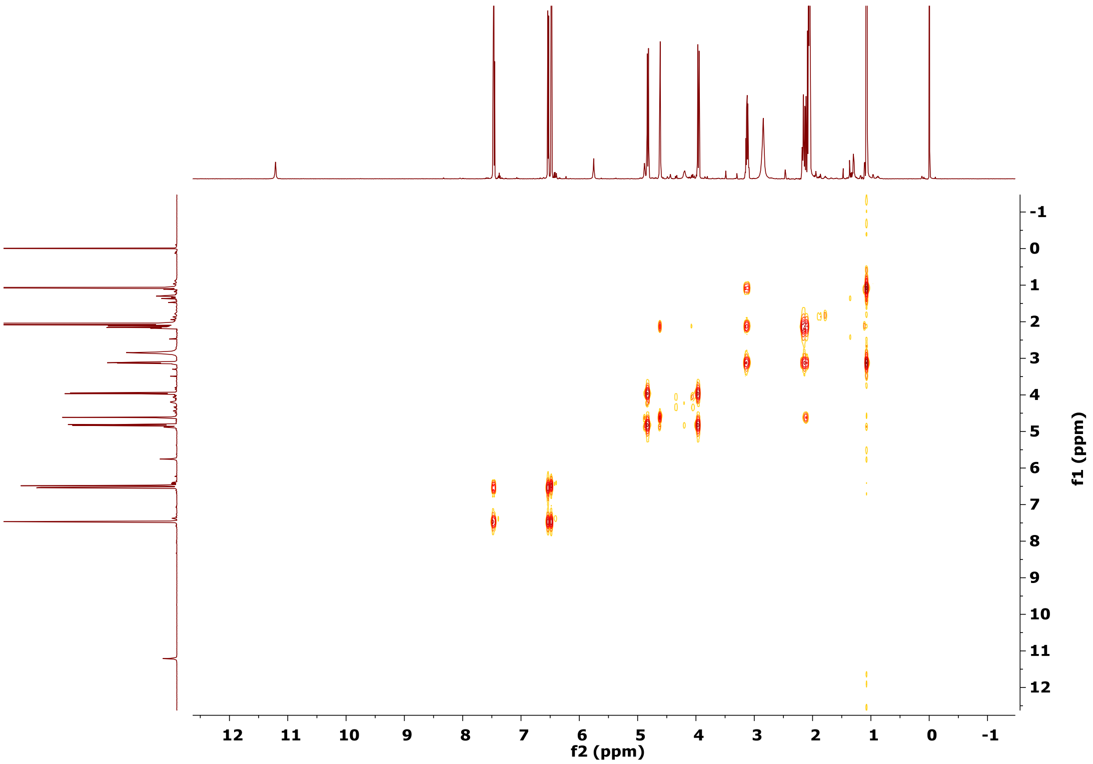

Figure S6. ^{1}H-^{1}H COSY spectrum of **1** in acetone-d_6.

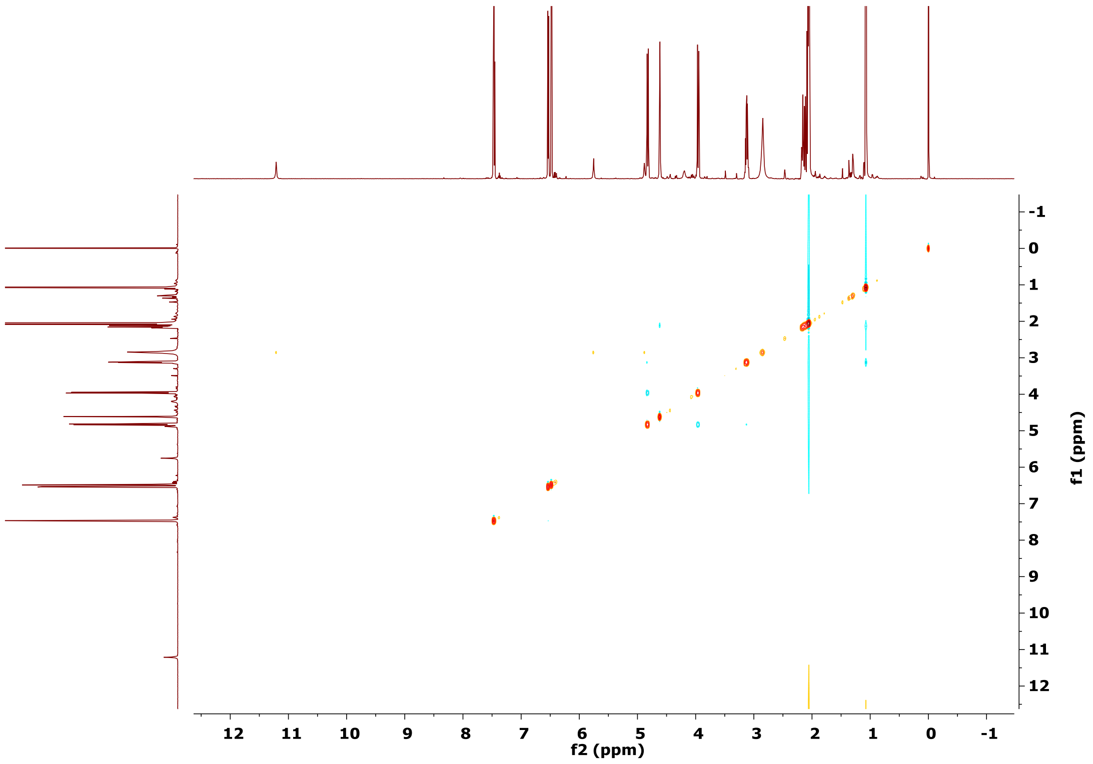

Figure S7. NOESY spectrum of **1** in acetone-d_6.

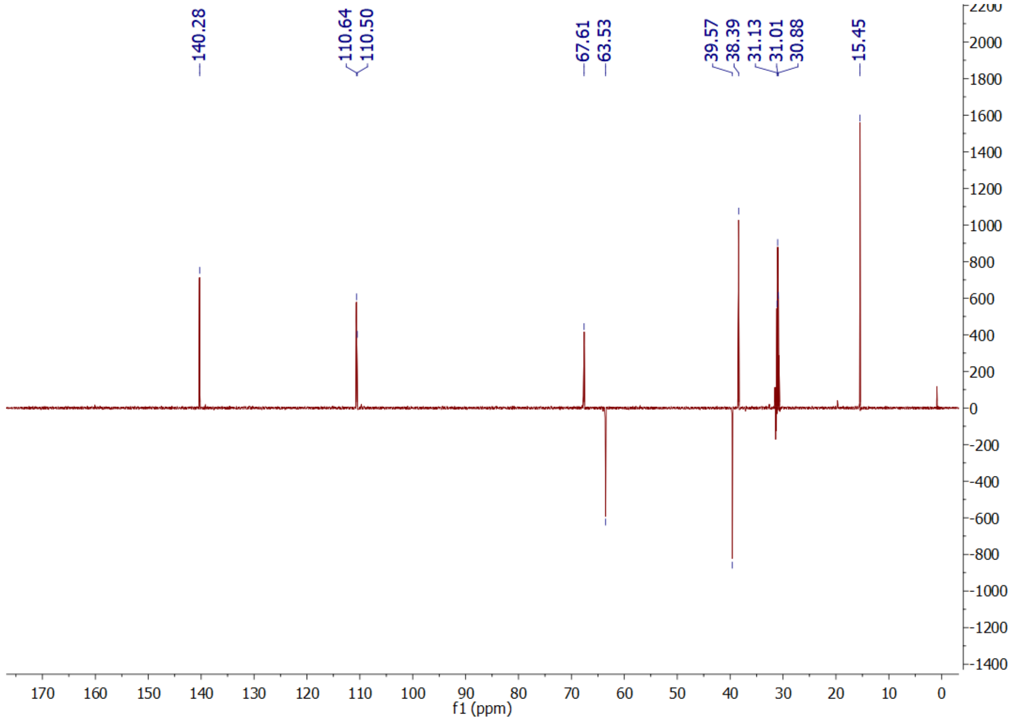

Figure S8. The DEPT spectrum of **1** in acetone-d_6.

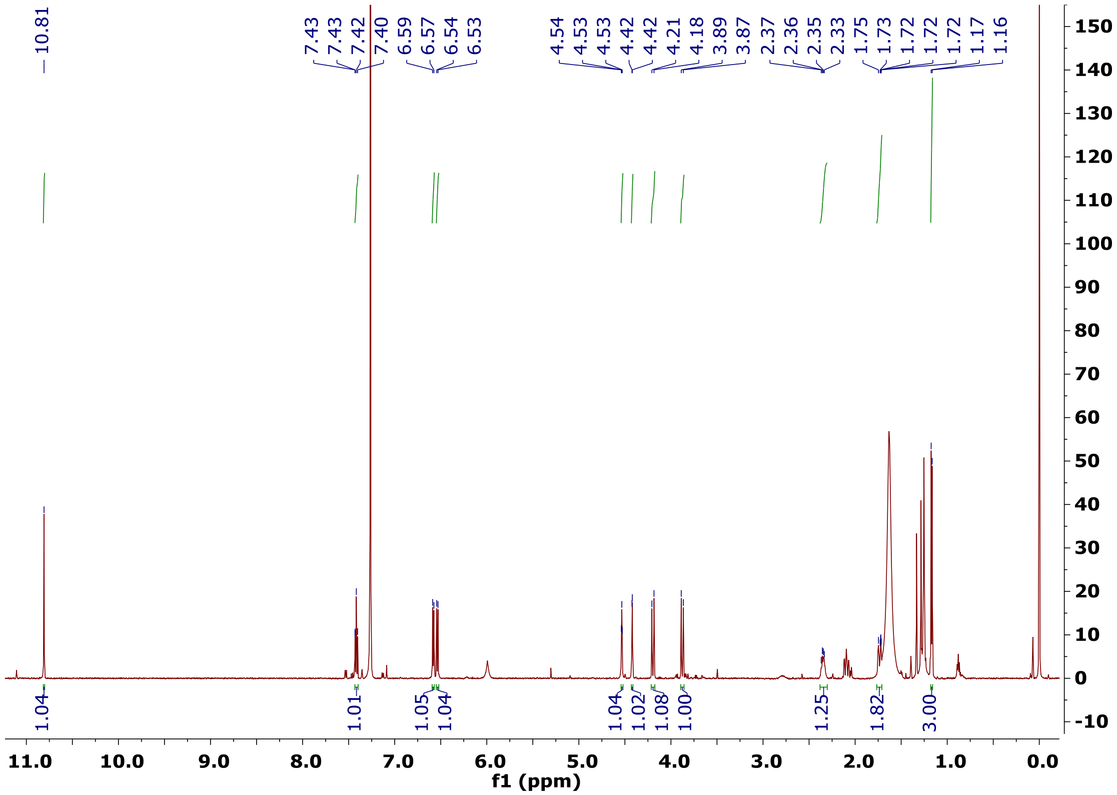

Figure S9. The ^{1}H-NMR spectrum of **2** ($CDCl_3$, 600 MHz).

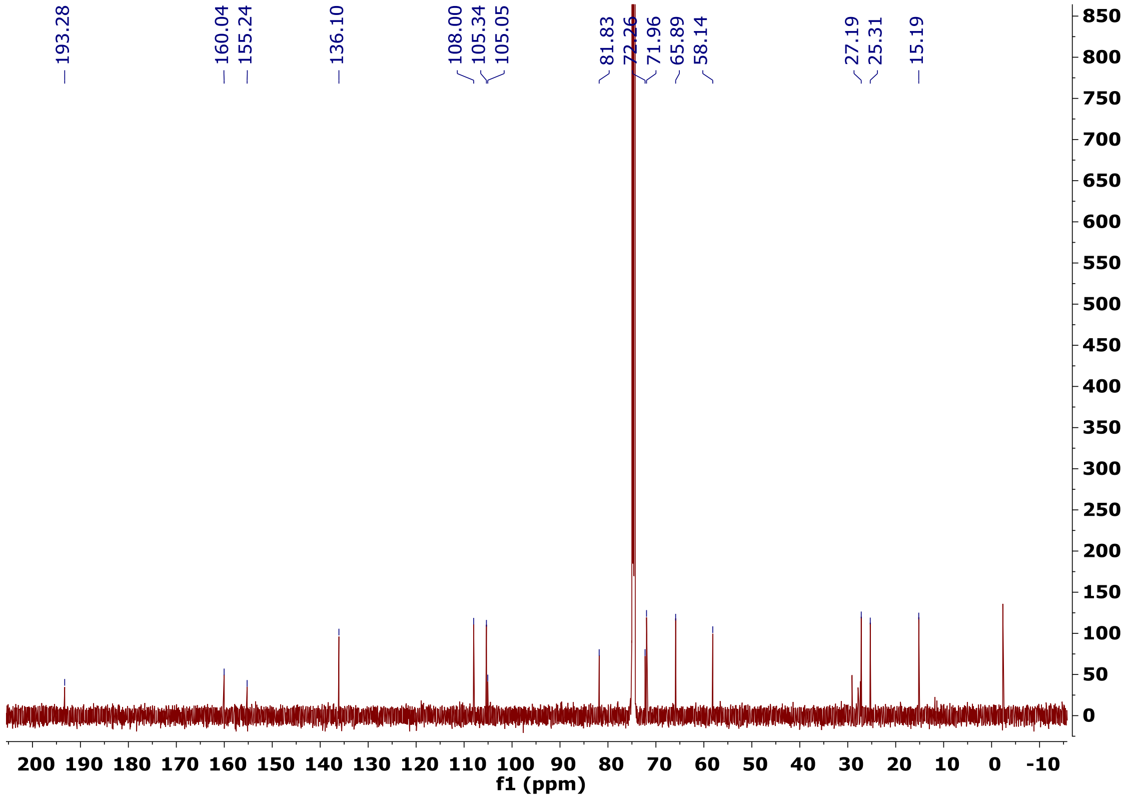

Figure S10. The ^{13}C-NMR spectrum of **2** ($CDCl_3$, 150 MHz).

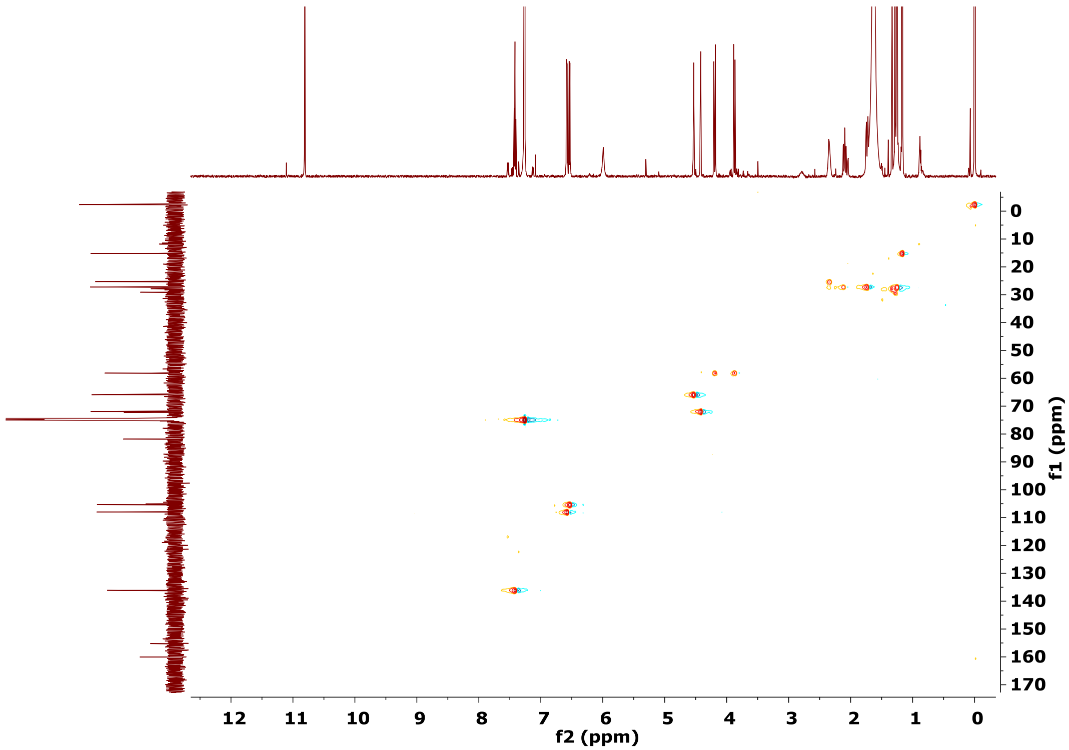

Figure S11. The HSQC spectrum of **2** in $CDCl_3$.

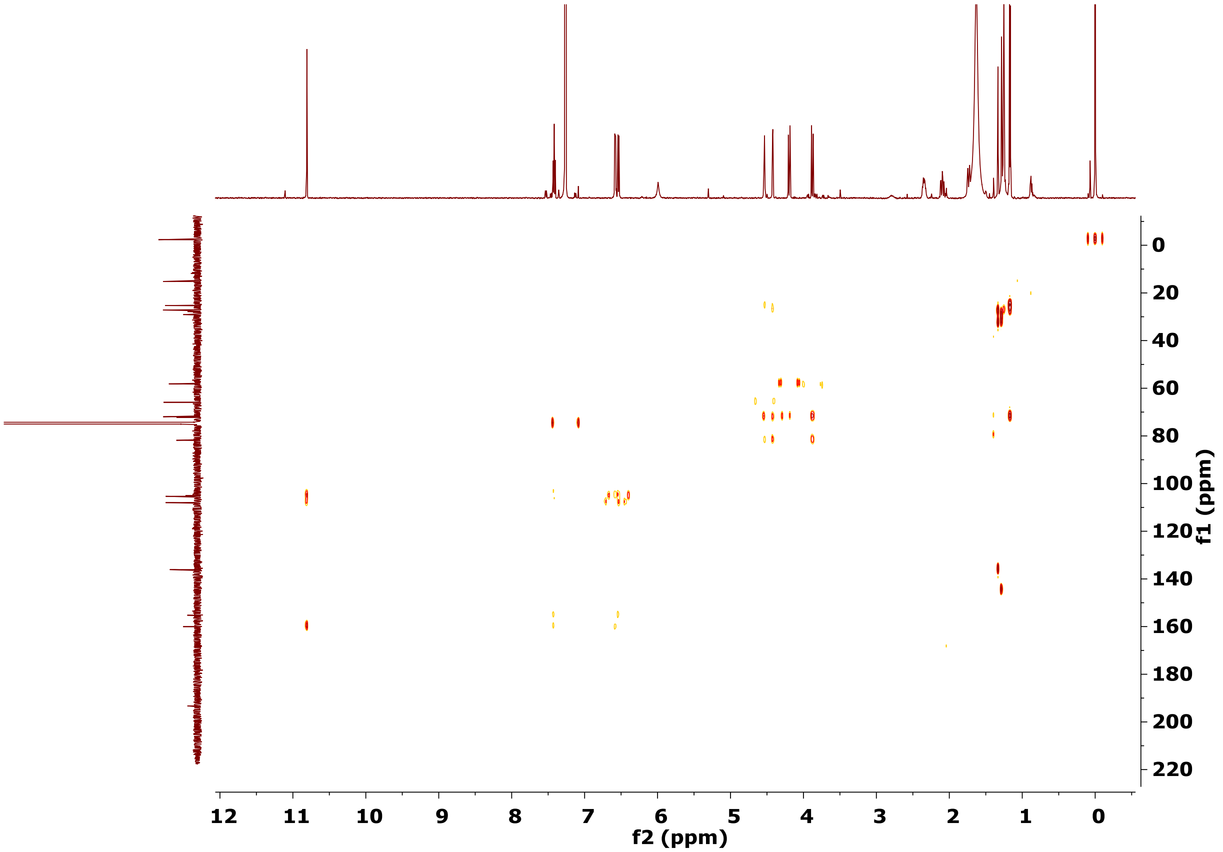

Figure S12. The HMBC spectrum of **2** in $CDCl_3$.

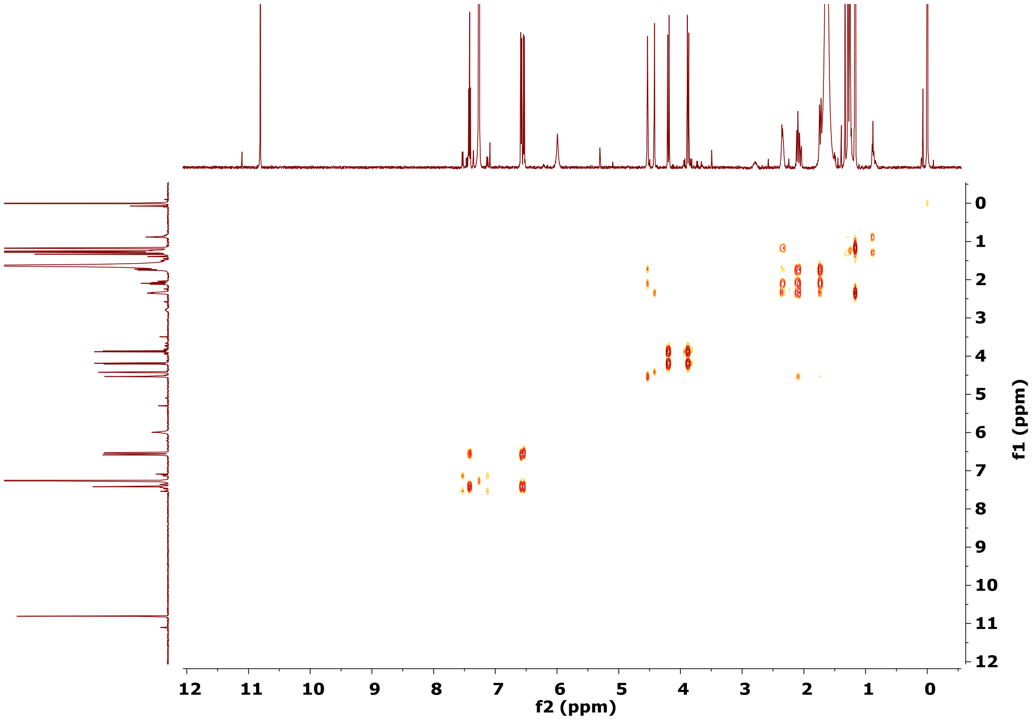

Figure S13. The ^1H-^1H COSY spectrum of **2** in $CDCl_3$.

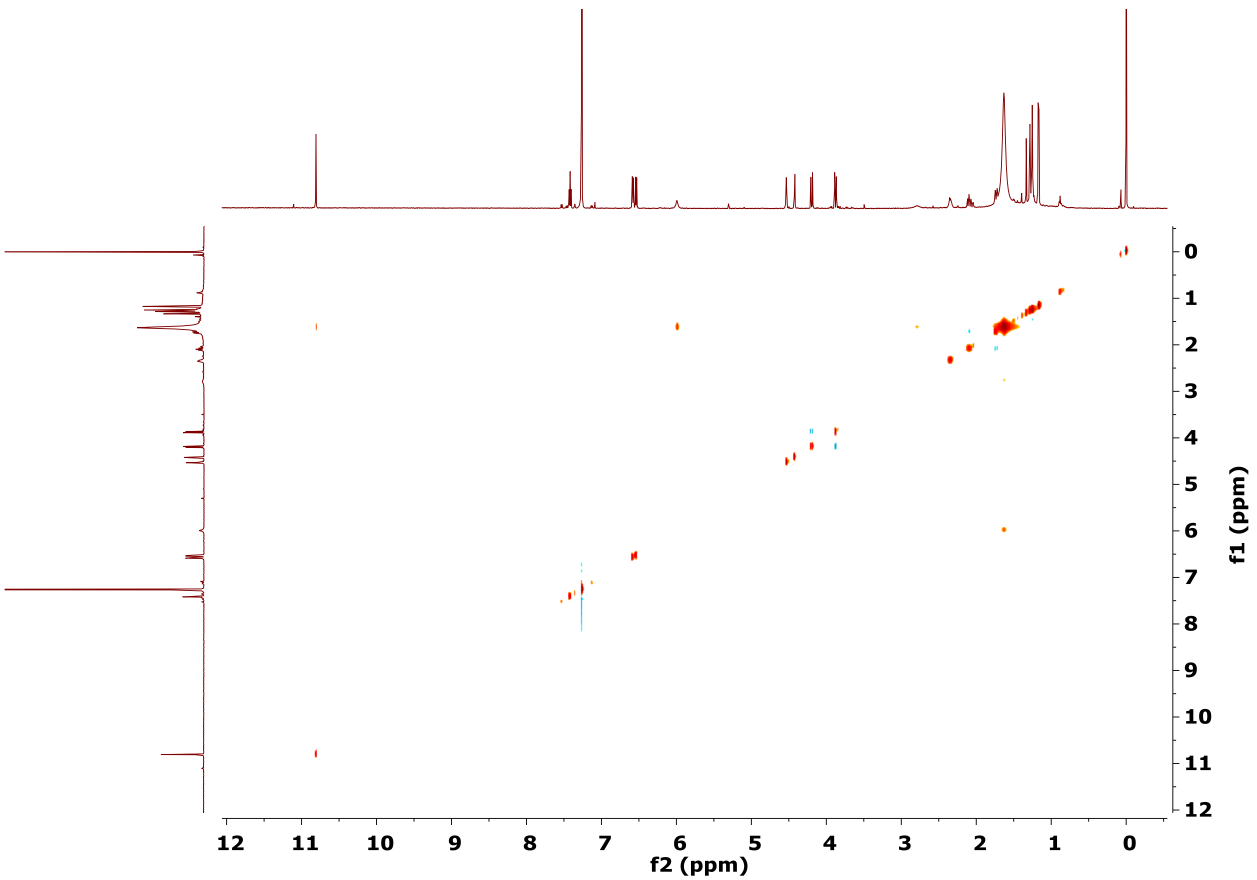

Figure S14. The NOESY spectrum of **2** in $CDCl_3$.

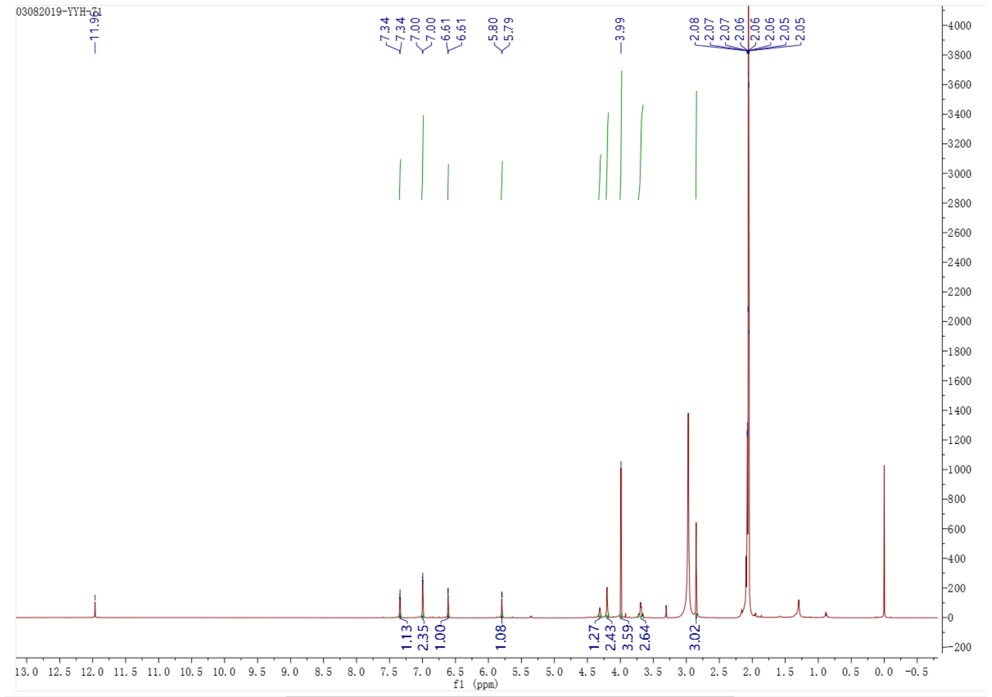

Figure S15. ^{1}H NMR spectrum of **3** in acetone-d_6 (600 MHz)

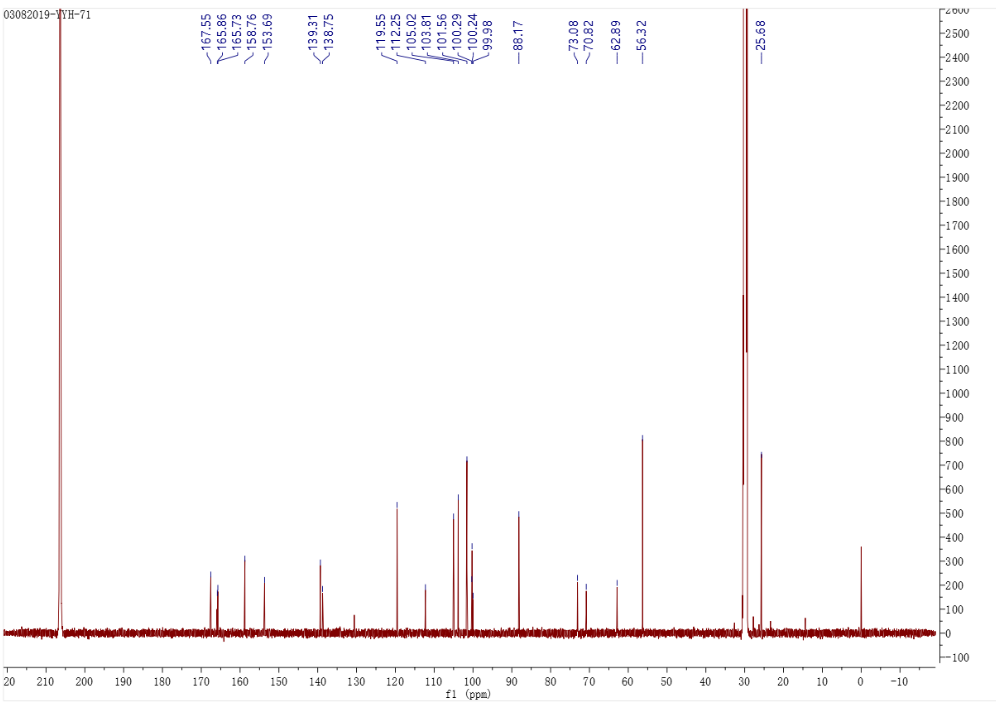

Figure S16. ^{13}C NMR spectrum of **3** in acetone-d_6 (600 MHz)

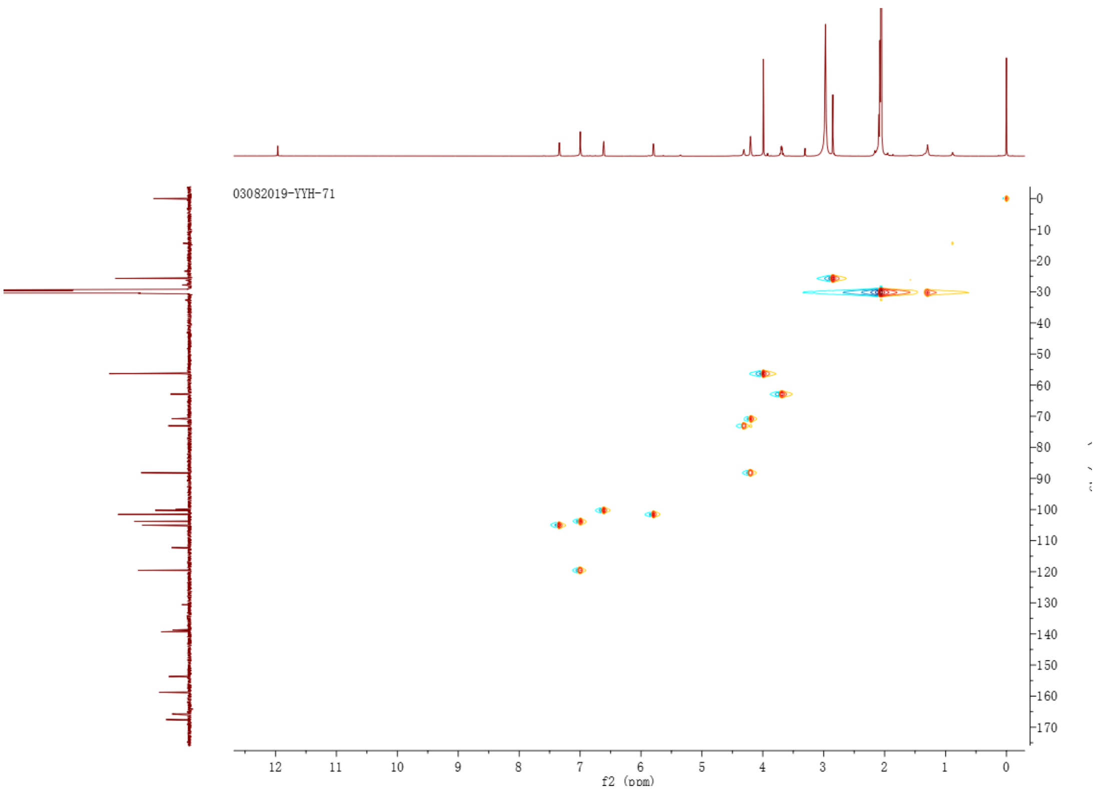

Figure S17. HSQC spectrum of **3** in acetone-d_6 (600 MHz)

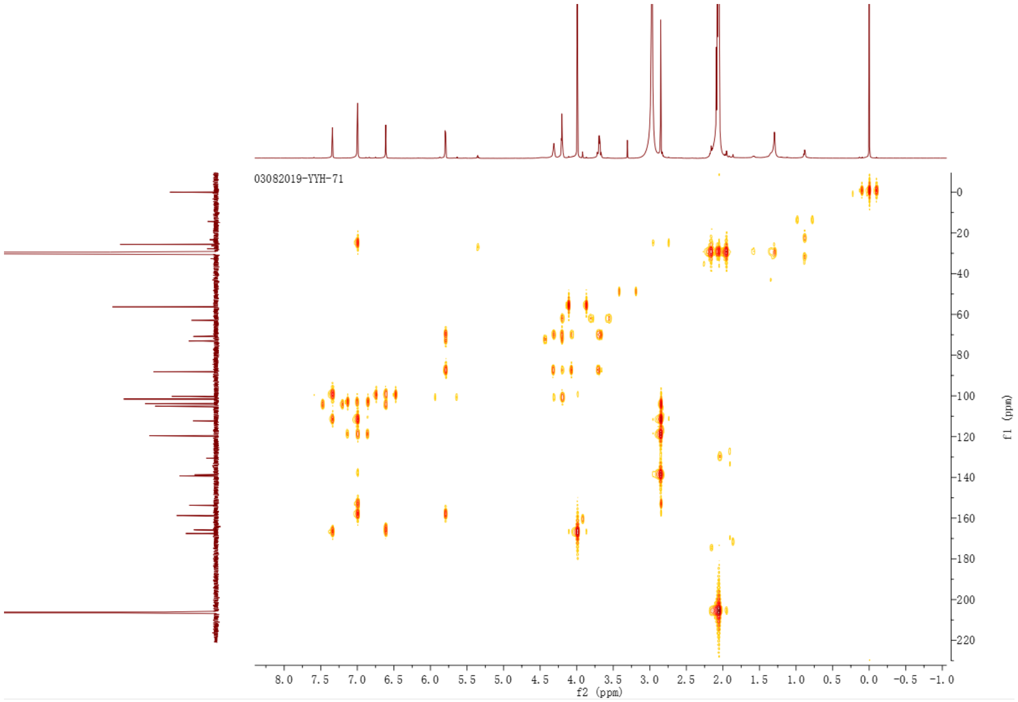

Figure S18. HMBC spectrum of **3** in acetone-d_6 (600 MHz)

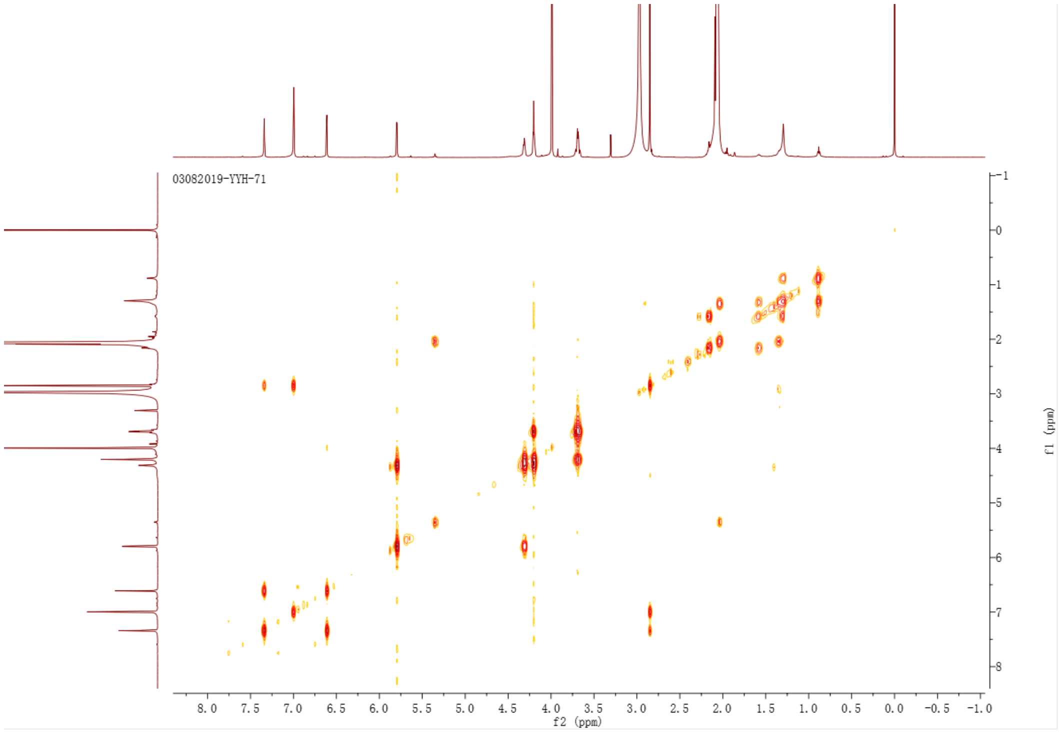

Figure S19. ^{1}H-^{1}H COSY spectrum of **3** in acetone-d_6 (600 MHz)

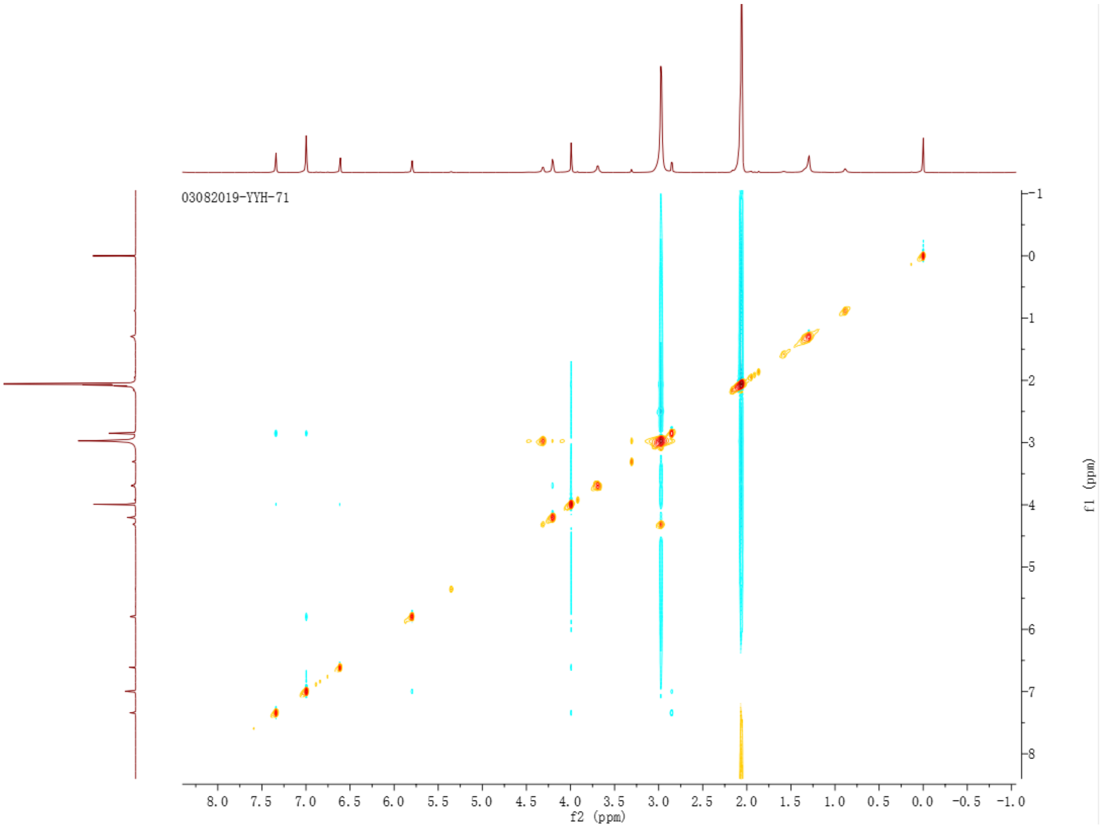

Figure S20. NOESY spectrum of **3** in acetone-d_6 (600 MHz)

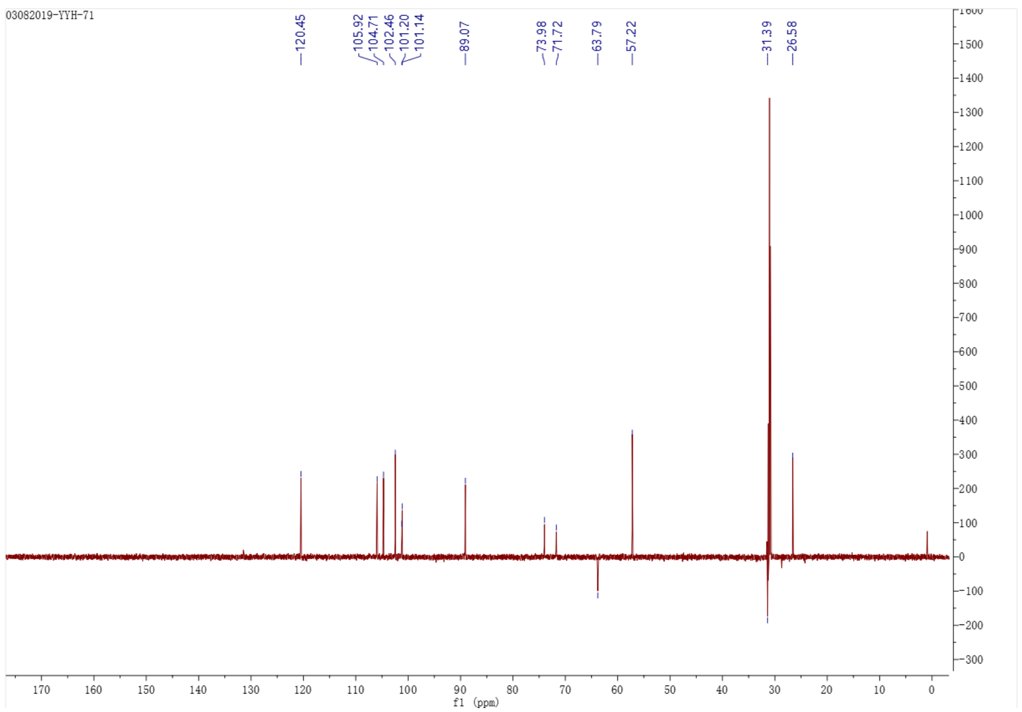

Figure S21. DEPT135 spectrum of **3** in acetone-d_6 (600 MHz)

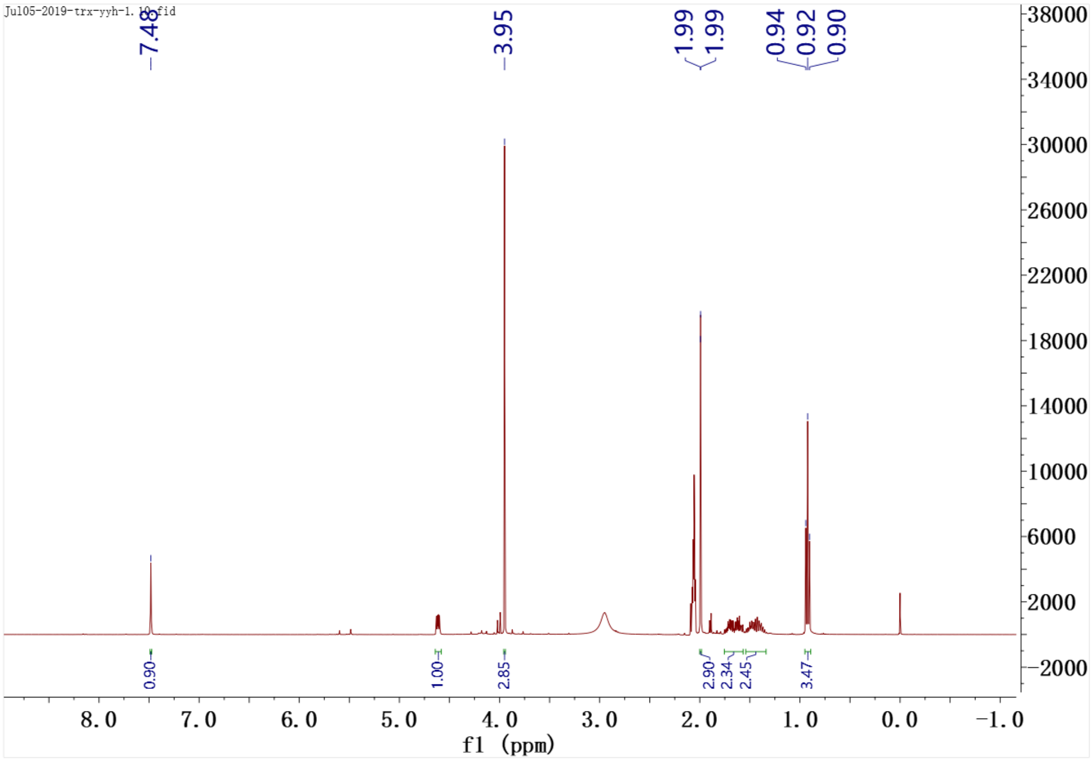

Figure S22. ^{1}H NMR spectrum of **4** in acetone-d_6 (400 MHz)

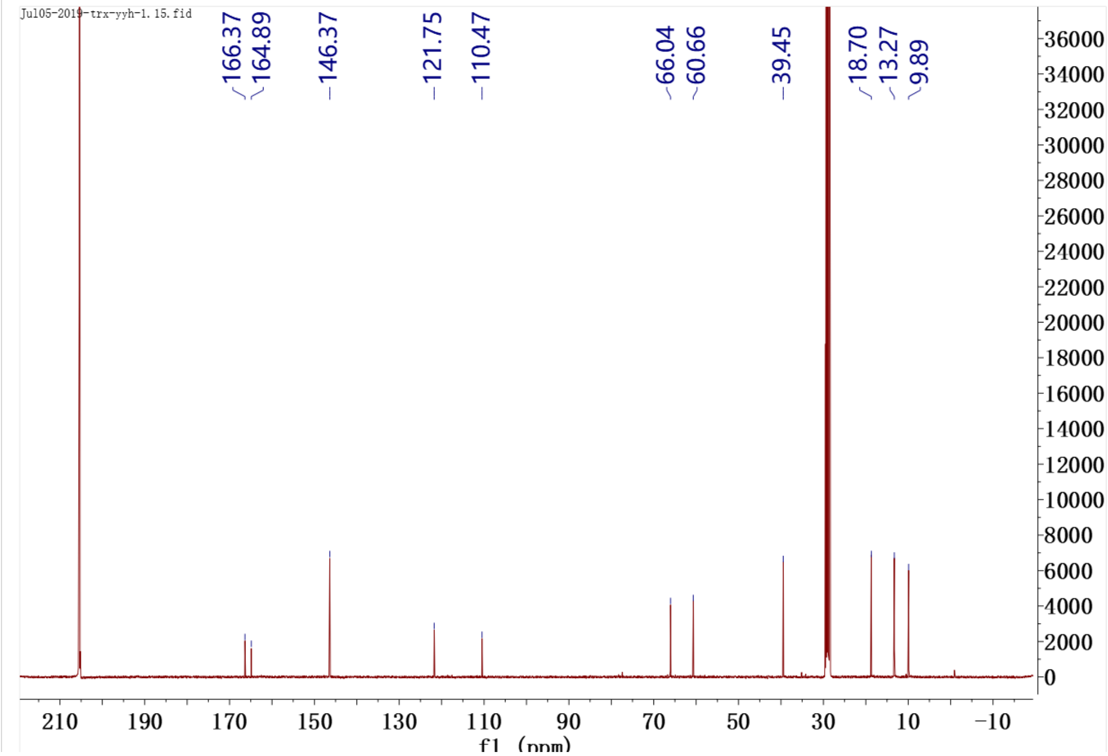

Figure S23. ^{13}C NMR spectrum of **4** in acetone-d_6 (100 MHz)

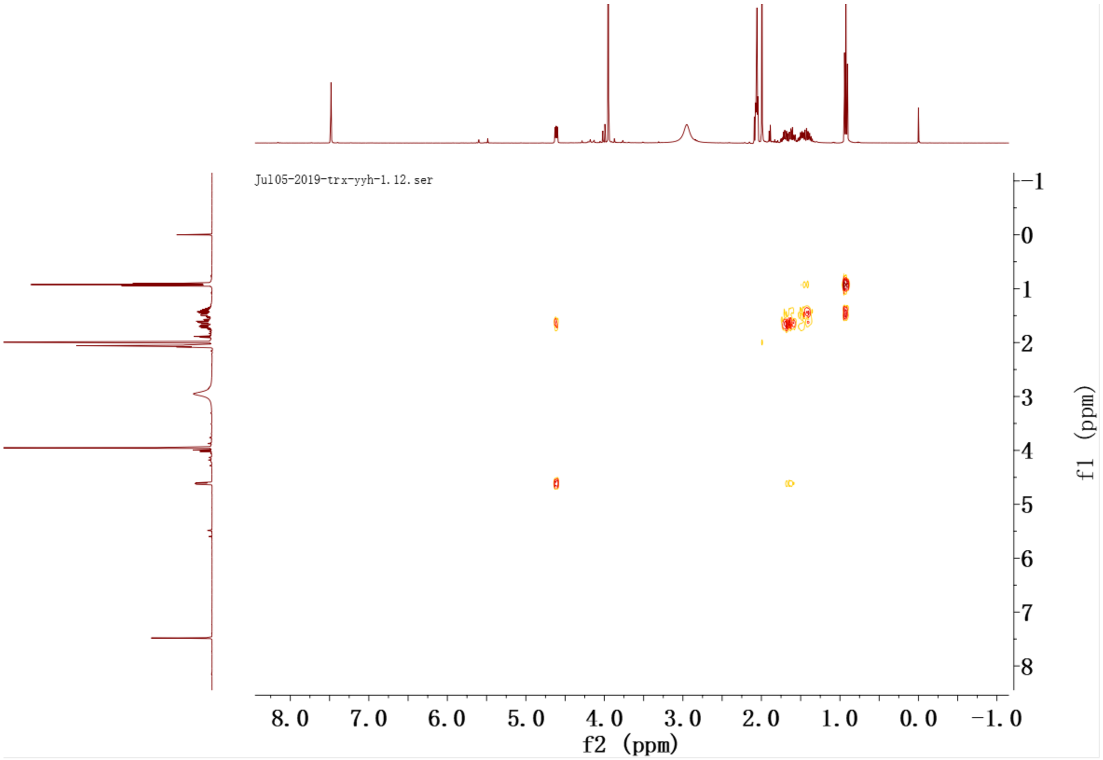

Figure S24. ^{1}H-^{1}H COSY spectrum of **4** in acetone-d_6 (400 MHz)

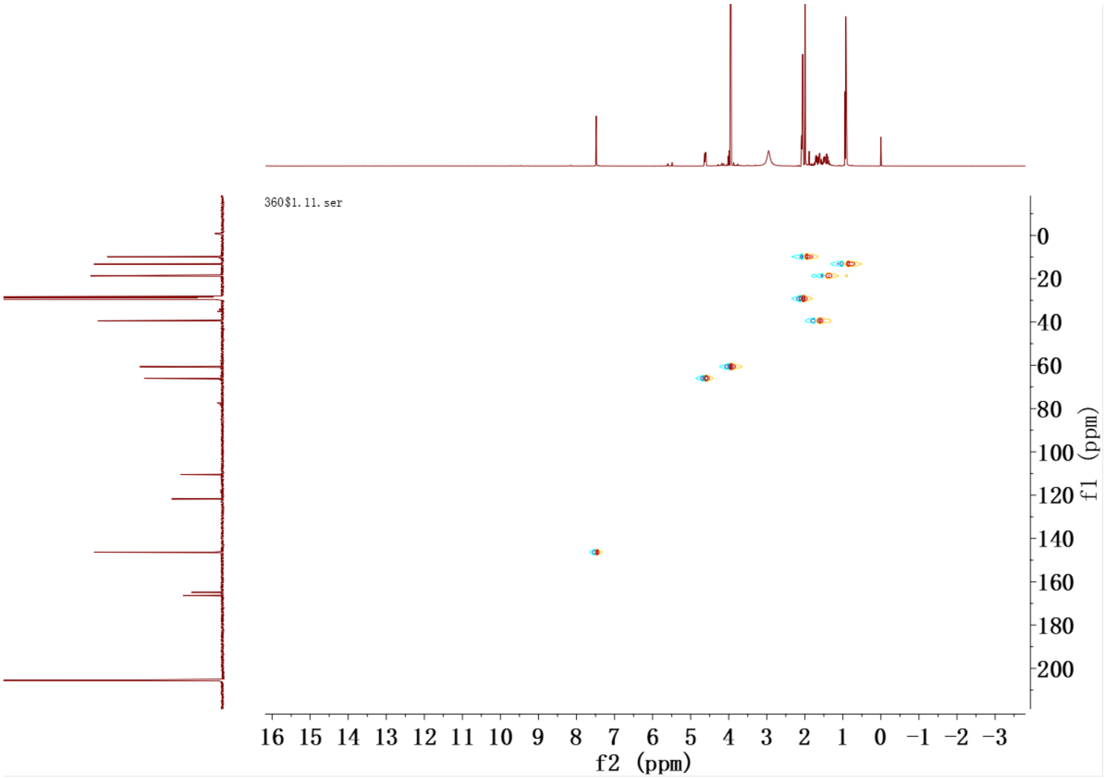

Figure S25. HSQC spectrum of **4** in acetone-d_6 (400 MHz)

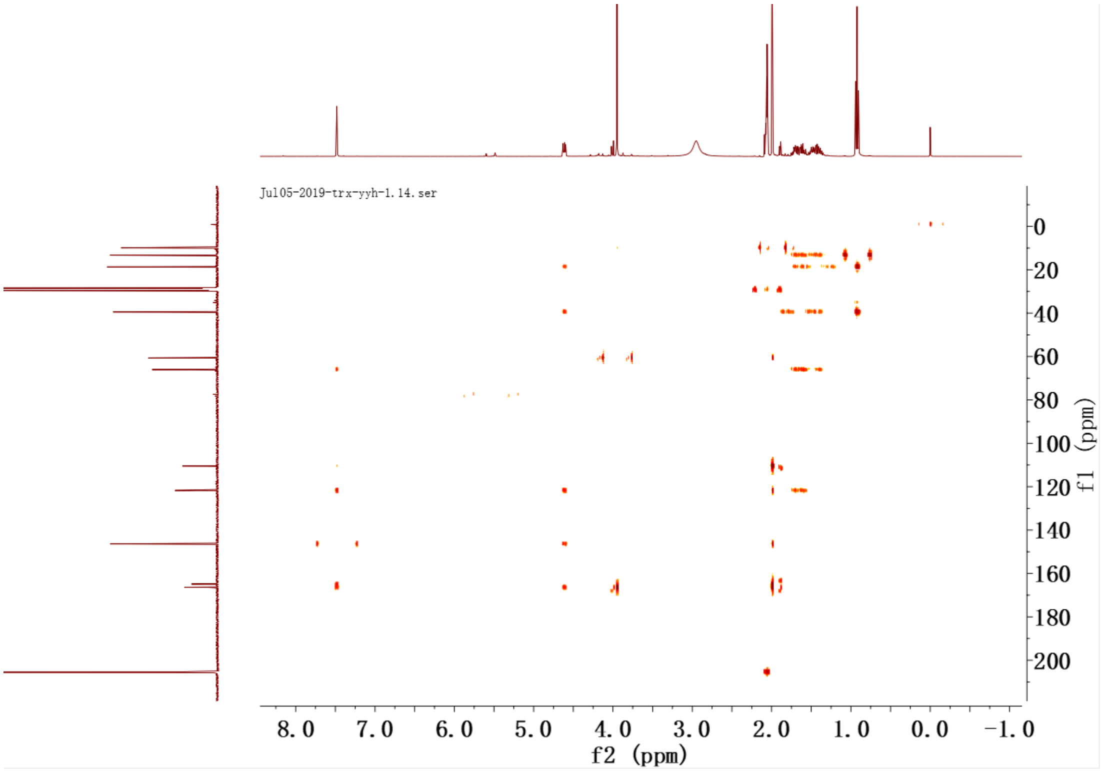

Figure S26. HMBC spectrum of **4** in acetone-d_6 (400 MHz)

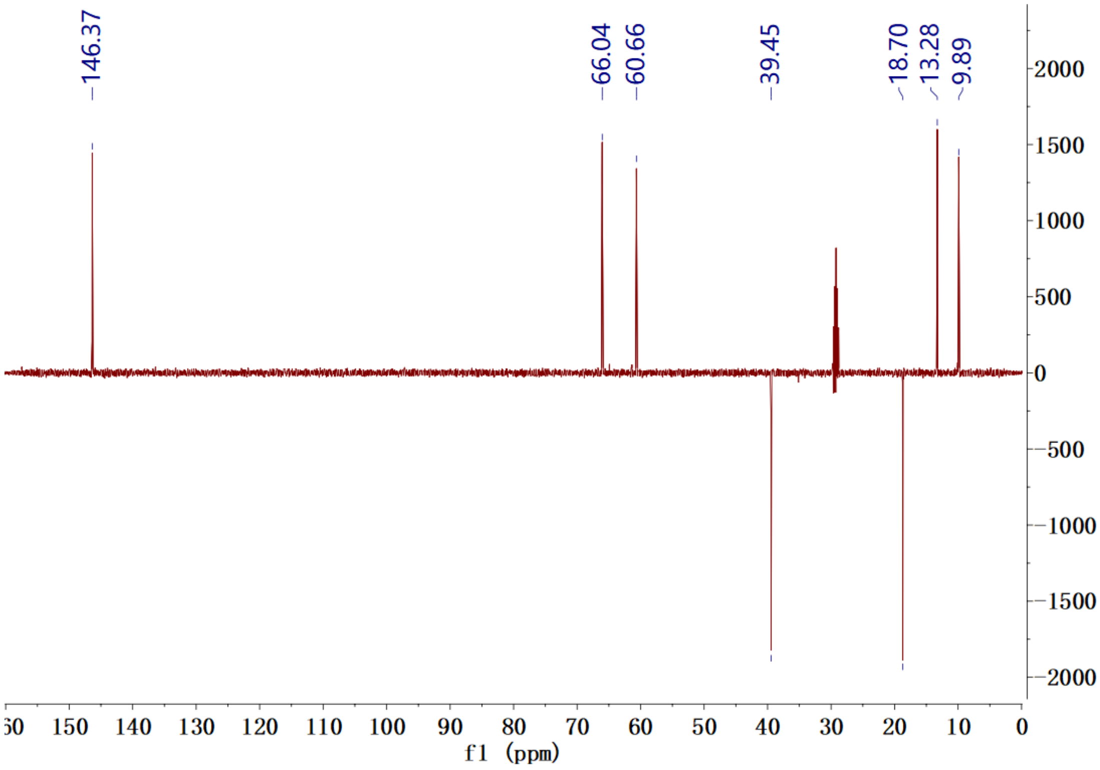

Figure S27. DEPT135 spectrum of **4** in acetone-d_6 (400 MHz)

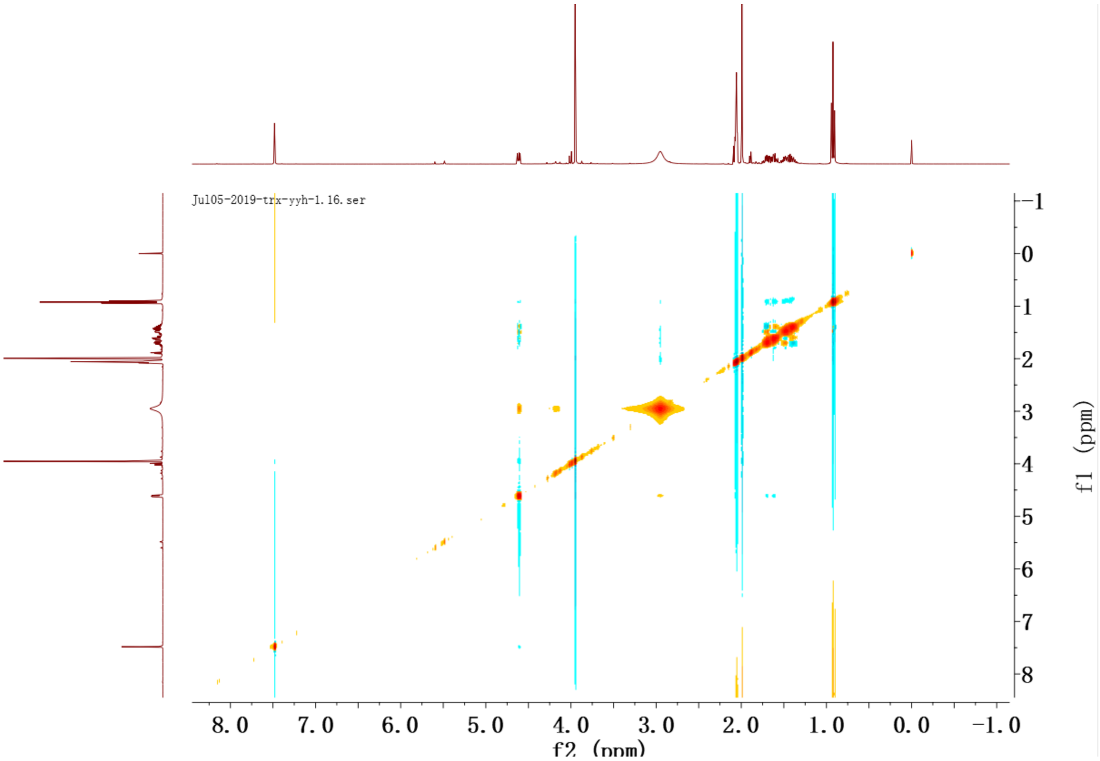

Figure S28. NOESY spectrum of **4** in acetone-d_6 (400 MHz)

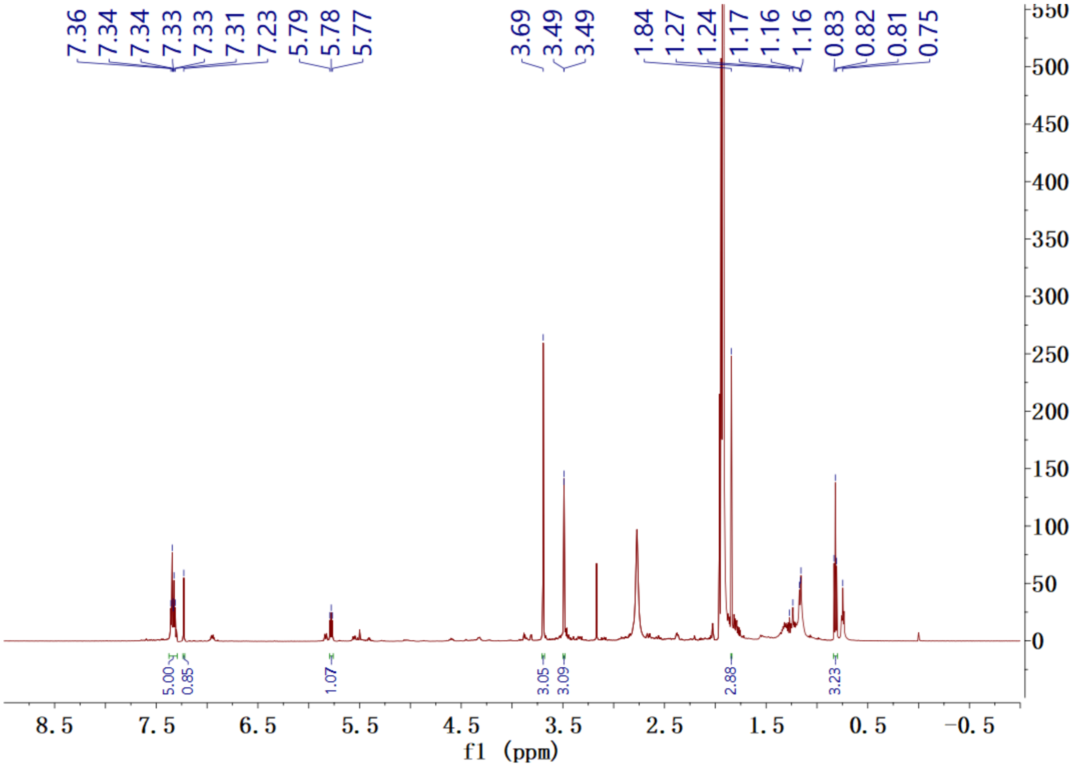

Figure S29. ^{1}H NMR of *S*-MTPA spectrum of **4** in acetone-d_6 (600 MHz)

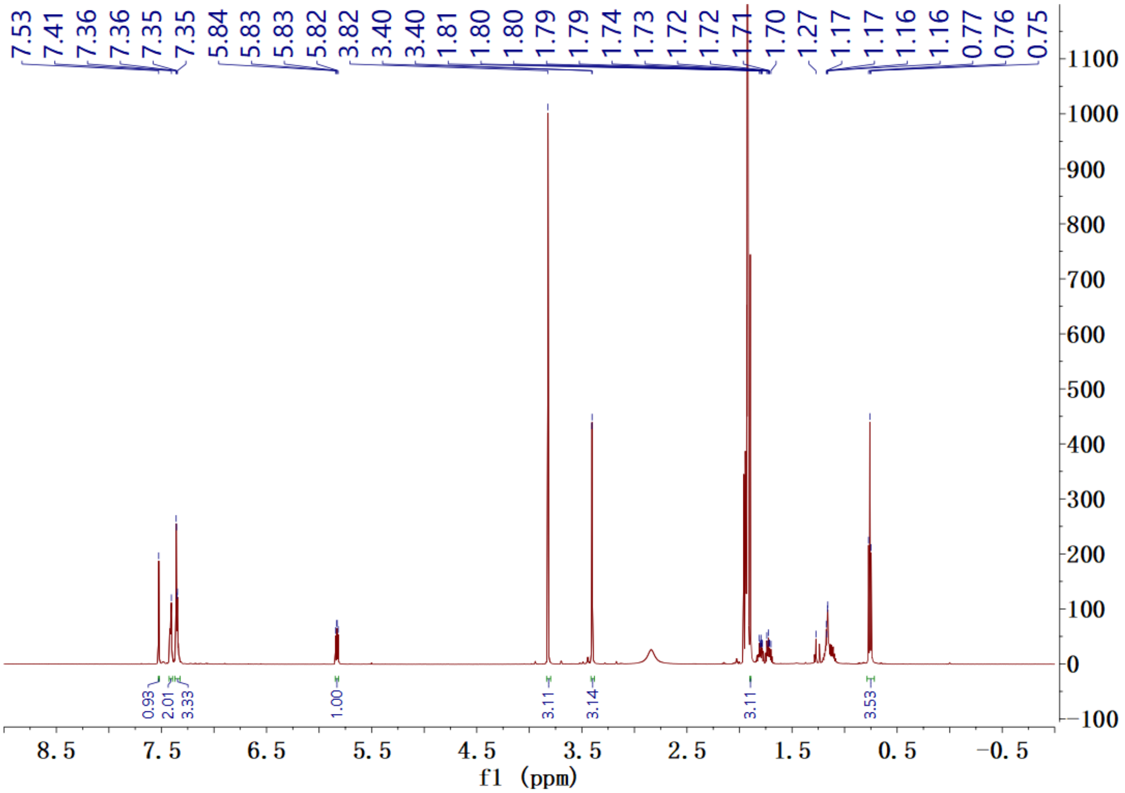

Figure S30. ^{1}H NMR of *R*-MTPA spectrum of **4** in acetone-d_6 (600 MHz)
